# 

**Published:** 1882-04

**Authors:** 


APRIL, 1882.—CONTENTS. PAGE

ORIGINAL COMMUNICATIONS—Tuberculosis in Men and Animals. By Thomas E. Satterthwaite, M.D. . . . 101
Observations on the Development of Special Senses. By John Dean Caton LL. D. . . . 109
Foreign Bodies in the Anterior Chamber of the Eye. By William Oliver Moore M. D. . . . 115
Pathology of the Nutritive Changes in the Cartilages and of Acute Synovitis. By Wm. Henry Porter, M.D., D.V.S. . . . 119
Some Diseases of Goats. By A. J. Sewell, V. S. . . . 130
The Physiology of Digestion in Ruminants, with Practical Remarks By Henry C. Slee. . . . 136
The Teeth of the Horse and their Diseases. By R. Jennings, V. S . . . 143
Breeding of Elephants in Captivity. By George Arstingstall, . . . 146
EDITORIAL—Tuberculosis in the Central Organs of the Nervous System in Cows—The Diseases to which the Domestic Animals are Most Subject—Veterinary Surgical Notes—Some Curious Things that Happen to our Domestic Animals—Some Anomalies of the Circulatory Organs—Diseases of Elephants—The Treatment of Scratches or Grease—Notices—Notice of Advertisements—Acknowledgement . . . 154–160
CASE DEPARTMENT—Epithelial Carcinoma of the Left Superior Maxilla, with Secondary Deposits in the Lungs—Rumenotomy—Reticulated Round-Cell Sarcoma of the Mediastinum, with Secondary Deposits in the Liver—Strongylus, Seu Eustrongylus Gigas Ascaris Renalis—Ulcer of the Œsophagus, Possibly Cancerous in Nature—Merotomy—Tenatomy . . . 161–172
CORRESPONDENCE . . . 172
REPORTS OF COMMENCEMENTS, SOCIETIES, ETC.—Columbia Veterinary College—American Veterinary College—Montreal Veterinary College—Ontario Veterinary College—County Veterinary Medical Society of the State of New York—Farmers' and Dairymen's Association—New Society . . . 173–178
REVIEWS—The Horse in Motion, as Shown by Instantaneous Photography—Nineteenth Annual Report of the State Board of Agriculture of the State of Michigan—Transactions of the Wisconsin State Agricultural Society—The Breeder's Gazette—Books and Pamphlets Received . . . 179–185
PROGRESS OF VETERINARY SCIENCE—The Best Disinfecting Agents—Resuscitation of Frozen Animals—Milk Vein—Oil Meals—Pleuro-Pneumonia in Cattle—The Effects of Cod Liver Oil on Monkeys—The Vivisection Question in the German Parliament—A Simple Means of Detecting Trichinæ—Notes on Seven Fatal Cases of Hydrophobia—Hydrophobia: Its Successful Treatment—Vaccine Applied to Sheep—Pleuro-Pneumonia in Cattle—The Horse's Frog—A Remedy for the Cattle Plague—Texas Fever, or Splenic Fever—Diarrhœa amongst Calves—Lung Sickness of Cattle—Wyoming Cattle Law—Glanders . . . 186–193
NEWS AND MISCELLANY—Honors to a Veterinary Surgeon—New Veterinary School—Weight of a Cow's Brain—The Celebrated Hunting Dog Lincoln—Greenwood Cemetery Report—Under Perfect Control—Spanish Cattle—Vivisection Again—Bears of Berne—New Horse Disease in Philadelphia—Clydesdale Horses—Scarlet Fever among Horses—How Street Car Horses are Fed—Cattle Troughs and Infectious Diseases—Birth of an Elephant in London—Hints for Curing Balky and Unruly Horses—The Goat Society—Ostrich Farming—The Photographer of the Galloping Horse—More of the Cattle Plague—Burial-Grounds for Animals—Free-Martins—Monkeys . . . 194–202

